# D1 receptor‐mediated endogenous tPA upregulation contributes to blood‐brain barrier injury after acute ischaemic stroke

**DOI:** 10.1111/jcmm.15570

**Published:** 2020-07-06

**Authors:** Yan Wang, Xiaona Wang, Xinyu Zhang, Shuang Chen, Yanyun Sun, Wenlan Liu, Xinchun Jin, Guoqing Zheng

**Affiliations:** ^1^ Department of Cardiology the Second Affiliated Hospital and Yuying Children's Hospital of Wenzhou Medical University Wenzhou China; ^2^ Institute of Neuroscience The Second Affiliated Hospital of Soochow University Suzhou China; ^3^ The Central Laboratory Shenzhen Second People’s Hospital Shenzhen University 1st Affiliated Hospital Shenzhen University School of Medicine Shenzhen China; ^4^ Department of Human Anatomy, Histology and Embryology School of Basic Medical Sciences Advanced Innovation Center for Human Brain Protection Capital Medical University Beijing China

**Keywords:** blood‐brain barrier, dopamine receptor, endogenous tissue plasminogen activator, HIF‐1α, ischaemic stroke, tight junction proteins

## Abstract

Blood‐brain barrier (BBB) integrity injury within the thrombolytic time window is becoming a critical target to reduce haemorrhage transformation (HT). We have previously reported that BBB damage was initially damaged in non‐infarcted striatum after acute ischaemia stroke. However, the underlying mechanism is not clear. Since acute ischaemic stroke could induce a significant increase of dopamine release in striatum, in current study, our aim is to investigate the role of dopamine receptor signal pathway in BBB integrity injury after acute ischaemia using rat middle cerebral artery occlusion model. Our data showed that 2‐h ischaemia induced a significant increase of endogenous tissue plasminogen activator (tPA) in BBB injury area and intra‐striatum infusion of tPA inhibitor neuroserpin, significantly alleviated 2‐h ischaemia‐induced BBB injury. In addition, intra‐striatum infusion of D1 receptor antagonist SCH23390 significantly decreased ischaemia‐induced upregulation of endogenous tPA, accompanied by decrease of BBB injury and occludin degradation. More important, inhibition of hypoxia‐inducible factor‐1 alpha with inhibitor YC‐1 significantly decreased 2‐h ischaemia‐induced endogenous tPA upregulation and BBB injury. Taken together, our data demonstrate that acute ischaemia disrupted BBB through activation of endogenous tPA via HIF‐1α upregulation, thus representing a new therapeutic target for protecting BBB after acute ischaemic stroke.

## 1 INTRODUCTION

Ischaemic stroke is a leading cause of morbidity and mortality worldwide.^1^ The most feared complication in thrombolytic therapy for acute ischaemic stroke is haemorrhagic transformation (HT),^2^, ^3^, ^4^ which occurs when blood‐brain barrier (BBB) integrity is disrupted.^5^ BBB damage during the reperfusion stage has been a key focus for the past decades, because HT and oedema will not occur if ischaemic brain is not reperfused.^6^, ^7^ However, it is not well studied for the mechanism underlying the BBB injury within the thrombolytic time window. Accumulating evidences demonstrated that HT occurs after thrombolytic reperfusion^8^ or post‐endovascular treatment^9^ if the brain regions showed BBB damage during the acute stage and the BBB damage acute ischaemic stroke is emerging as both a predictor and a promising target for HT in clinic.^10^

We have previously checked the spatial and temporal change of BBB injury and brain tissue damage. Our results demonstrated that 2‐h ischaemia induced cortex and dorsal striatum infarction and BBB injury in non‐infarcted ventral striatum and pre‐optical area.^11^, ^12^ However, it is not clear why BBB injury was initially found in non‐infarcted area. It is noteworthy that our recent results showed that upregulated HIF‐1α in striatum played important role in BBB injury after acute ischaemic stroke.^13^, ^14^ In addition, HIF‐α has been shown to regulate dopamine release^15^ which has been shown to play an important role in ischaemic stroke‐induced brain damage.^16^ Acute ischaemic stroke‐induced dopamine release in the striatum was greater (400‐fold over pre‐ischaemic level) than that in the cortex (12‐fold over pre‐ischaemic level).^17^ Dopamine has been shown to be involved in acute 3‐nitropropionic acid‐induced striatal astrocytic cell death and dysfunction of the BBB.^18^ However, it is not clear whether ischaemia‐induced dopamine release could induce BBB damage and the underlying mechanism is not clear.

It has been reported that D1 receptor agonist SKF38393 significantly increased endogenous tissue plasminogen activator (tPA) activation in ventral striatum and activation of post‐synaptic dopamine D1 receptors by systemic administration of morphine or methamphetamine promoted the release of tPA via protein kinase A (PKA) signalling.^19^ In addition, it has been demonstrated that intra‐nigral injection of tPA disrupted BBB^20^ and tPA can increase the permeability of BBB via the low density lipoprotein receptor‐related protein,^2^ via plasmin‐mediated activation of the Rho kinase pathway in astrocytes^21^ and via activation of platelet‐derived growth factor C (PDGF‐CC).^22^

In the current study, we aimed to test the hypothesis that ischaemia disrupted BBB through activation of endogenous tPA via HIF‐1α upregulation‐induced dopamine receptor activation.

## 2 MATERIALS AND METHODS

### 2.1 Animal model of focal cerebral ischaemia

Sprague Dawley male rats (50‐55 days, RRID: RGD_70508) weighing 270‐290 g were ordered from SLAC Company (Shanghai, China). They were housed three per cage in a temperature‐ and humidity‐controlled *vivarium* on a reversed 12 hour‐12 hour light‐dark cycle. Rats had unlimited access to water and food. The animal procedures were in accordance with the Soochow University Committee on Animal Care (approval #SYXK (SU) 2017‐0043). All animal experiments were performed were in accordance with the guideline of NIH for the Care and Use of Laboratory Animals to minimize animal suffering and to reduce the number of animals. All animals were anaesthetized with isoflurane (#O2140, MAC 1.15) and were placed on a thermostatic blanket during MCAO.

Rats (n = 105) were subjected to 2‐hour middle cerebral artery occlusion (MCAO) using the intraluminal suture occlusion model, as we described previously.^23^ Rats housed in the same cage underwent the same manipulations. The MCAO rat model success rate is 100% without any accident of intracranial bleeding and no rats died because of stroke or surgical complications. Rats were killed with transcardially being perfused with ice‐cold PBS or 4% PFA after 2‐hour MCAO/reperfusion followed by quick removal of the brain.

### 2.2 Drug treatment

#### 2.2.1 Neuroserpin administration

To inhibit tPA activity, specific inhibitor neuroserpin (20 μmol/L, 3 μL, Cat#13014250; PeproTech Company (Rocky Hill, New Jersey, USA), dissolved in PBS) or vehicle was infused into striatum (AP −1.0, ML −3.0, DV −7.0) at a rate of 9 μL/h 30 minutes prior to the onset of ischaemia, after infusion the needle stayed for another 5 minutes before removal.^24^, ^25^ Simple randomization was employed to allocate rats (n = 14) to vehicle and neuroserpin group (n = 7/group).

#### 2.2.2 SCH23390 administration

To block interaction of dopamine with dopamine receptor 1 in early ischaemic BBB damage, the D1 receptor antagonist SCH23390 (1 mg/mL, 0.64 μL, Cat#D054; Sigma, St. Louis, MO, USA, dissolved in saline) or vehicle was infused into striatum (AP −1.0, ML −3.0, DV −7.0) immediately prior to the onset of ischaemia. After infusion, the needle stayed for another 5 minutes before removal.^26^ Simple randomization was employed to allocate rats (n = 24) to vehicle and SCH23390 group (n = 12/group).

#### 2.2.3 YC‐1 administration

YC‐1 (Cayman Chemical Company, Ann Arbor, Michigan, USA) is dissolved in a solution of 1% dimethyl sulphoxide (DMSO). Rats received YC‐1 (2 mg/kg) or vehicle via femoral vein at 24 hours and 30 minutes before ischaemia.^14^ Simple randomization was employed to allocate rats (n = 10) to vehicle and YC‐1 group (n = 5/group).

### 2.3 Evan's blue (EB) leakage detection

EB (Cat#E2199, Sigma, 2% w/v in PBS) was intravenously injected (3 mL/kg) through the left femoral vein immediately after MCAO as we described previously.^12^ All rats were given 10 minutes reperfusion for sufficient EB circulation to the ischaemic brain and less reperfusion‐induced BBB injury. The rat brain was quickly removed after the rat was transcardially perfused with ice‐cold PBS.

1. Spatial distribution of BBB injury could be observed by checking the EB leakage in ten consecutive 1‐mm‐thick coronary slices as we described previously.
2. EB leakage was also recruited to quantitate BBB disruption by measuring content in the non‐ischaemic and ischaemic brain tissue as we reported.^23^
3. EB leakage combined with occludin IHC was used to check whether occludin degradation in the area of BBB damage. The slide was scanned in a LSM700 microscope (Carl Zeiss, Weimar, Germany), and the coronal image was reconstructed using adobe photoshop. EB appeared as red fluorescence on brain sections with excitation wavelength of 542 nm and a 560‐nm long‐pass filter for collecting fluorescence emission.^27^

### 2.4 Evaluation of BBB integrity by immunoglobulin G leakage

Blood‐brain barrier integrity can also be evaluated by checking immunoglobulin G (IgG) leakage as we reported previously.^12^ Briefly, After 20 minutes fixation with 4% PFA for at room temperature, the 20‐μm‐thick section was stained with Cy3‐conjugated Affinity Pure Goat anti‐Rat IgG (1:400, RRID: AB_2632462; Jackson ImmunoResearch Laboratories Inc., West Grove, Pennsylvania, USA) for 2 hours, followed by mounted with a glass coverslip. The coronal image was achieved from a LSM700 microscope (Carl Zeiss).

### 2.5 In situ *tPA casein zymography*

The rat was transcardially perfused with PBS, followed by quick removal of the brain, freeze in OCT (Sakura Finetechnical, Tokyo, Japan) and store at −80°C. Cryosections (20 μm) were analysed for in situ proteinase activity as described previously.^19^ In brief, 100 μl overlays of 1% agarose in PBS containing 10 μg/mL of BODIPY TR‐X FL casein (Molecular Probes, #E6638, Invitrogen, CA, USA) and 5 mmol/L EDTA with plasminogen (#P7999; Sigma), were added to pre‐warmed tissue and sealed under glass coverslips. After 2‐hours incubation at 37°C, image of casein fluorescence was achieved with a microscope (model Axioskop; Carl Zeiss Vision).

### 2.6 Immunostaining

The 20‐µm‐thick cryosection from each group was fixed with 4% PFA for immunostaining analysis for occludin, as we reported previously.^23^ Briefly, tissue were pre‐incubated in PBS containing 0.1% Triton X‐100, 1% BSA and 5% goat serum for 1 hour at room temperature to block non‐specific binding sites. The section was then incubated overnight with primary antibody of anti‐occludin (1:150, RRID: AB_2533977; Invitrogen) at 4°C followed by incubation with 488‐conjugated secondary antibody (anti‐rabbit, 1:800, RRID: AB‐143165) for 2 hours at room temperature. Images were achieved from the region and the mirrored region of ischaemic and the non‐ischaemic hemisphere, respectively, under LSM 700 confocal laser‐scanning microscope (Zeiss).

### 2.7 Western blot analysis for occludin, tPA and HIF‐1α

The experiment was done as we have described previously.^28^ Tissue in the regions of interest (ROI 1, tissue damage area; ROI 2, BBB damage area) of ischaemic and mirror non‐ischaemic hemisphere was collected as we described.^14^ Briefly, protein samples were electrophoresed in SDS‐PAGE acrylamide gels and transferred onto nitrocellulose membranes (Bio‐Rad, Hercules, California, USA). The membranes were incubated for 60 minutes in 5% non‐fat milk, followed by incubation overnight at 4°C with primary antibodies against occludin (1:300, Invitrogen), HIF‐1α (1:300; Novus, Centennial, Colorado, USA) and tPA (1:500; Abcam). After incubation for another 60 minutes with corresponding HRP‐conjugated anti‐rabbit or anti‐mouse antibodies (1:3000, RRID:AB_2734136 or #BA1050, Boster) at room temperature, the membranes were developed with the SuperSignal West Pico HRP substrate kit (#WBKLS0500; Pierce, Rockford, Illinois, USA) and photographed on a Gel DOC^TM^ XR^+^ image station (Bio‐Rad). Protein band intensities were quantitated after normalization to β‐actin (Cat#M1210‐2) or β‐tublin (Cat#0807‐2).

### 2.8 Statistical analysis

All data shown were analysed using Power Analysis and Sample Size (PASS) software (version 17.0) and were graphed using GraphPad Prism software (version 5). The data are presented as mean ± SEM. The number of independent experiments was shown in the figure legends. The normality of the data was confirmed by the Shapiro‐Wilk test. No test for outliers was conducted on the data. Comparisons among two or four groups were carried out by one‐way ANOVA with Bonferroni's multiple comparisons post hoc test. A value of *P* < 0.05 was considered statistically significant. During sample preparation and analysis, the investigators were blinded to the experimental groups.

## 3 RESULTS

### 3.1 Spatial distribution of endogenous tPA after acute ischaemia stroke

**FIGURE 1:**
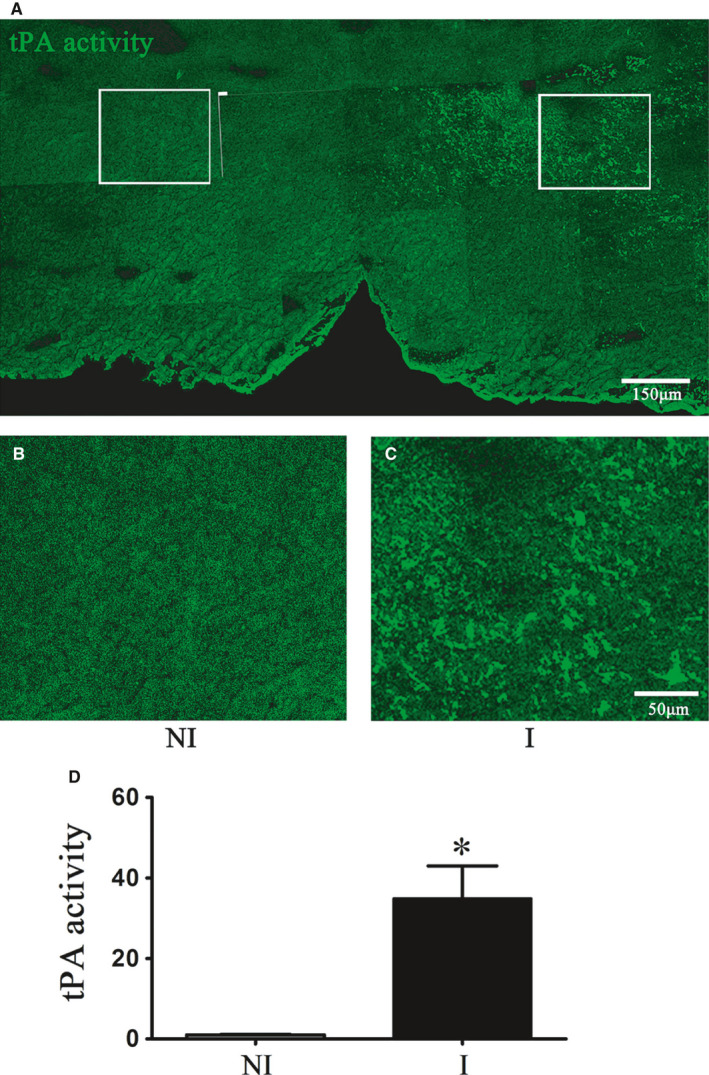
Spatial distribution of endogenous tPA after 2‐h MCAO. A, Representative representation of tPA activity showed increased tPA in the ventral striatum and preoptic area of ischaemic hemisphere after 2‐h MCAO detected by in situ tPA casein zymography. Corresponding enlarged view of the box in left (B) and right (C) side of (A). tPA activity in non‐ischaemic and ischaemic hemisphere was quantitated and expressed as relative fluorescence intensity after normalization to the non‐ischaemic (NI) hemisphere, n = 3/group (D)

Endogenous tPA has been shown to modulate BBB integrity.^29^ In situ tPA Casein zymography was used to detect spatial distribution of tPA activity after 2‐hour ischaemia, and our data demonstrated that 2‐hour ischaemia induced significant increase of tPA activity (green) in ventral striatum and pre‐optical area of ischaemic hemisphere (Figure 1A‐D).

### 3.2 Effect of intra‐striatum infusion of neuroserpin on 2‐h MCAO‐induced BBB damage

**FIGURE 2:**
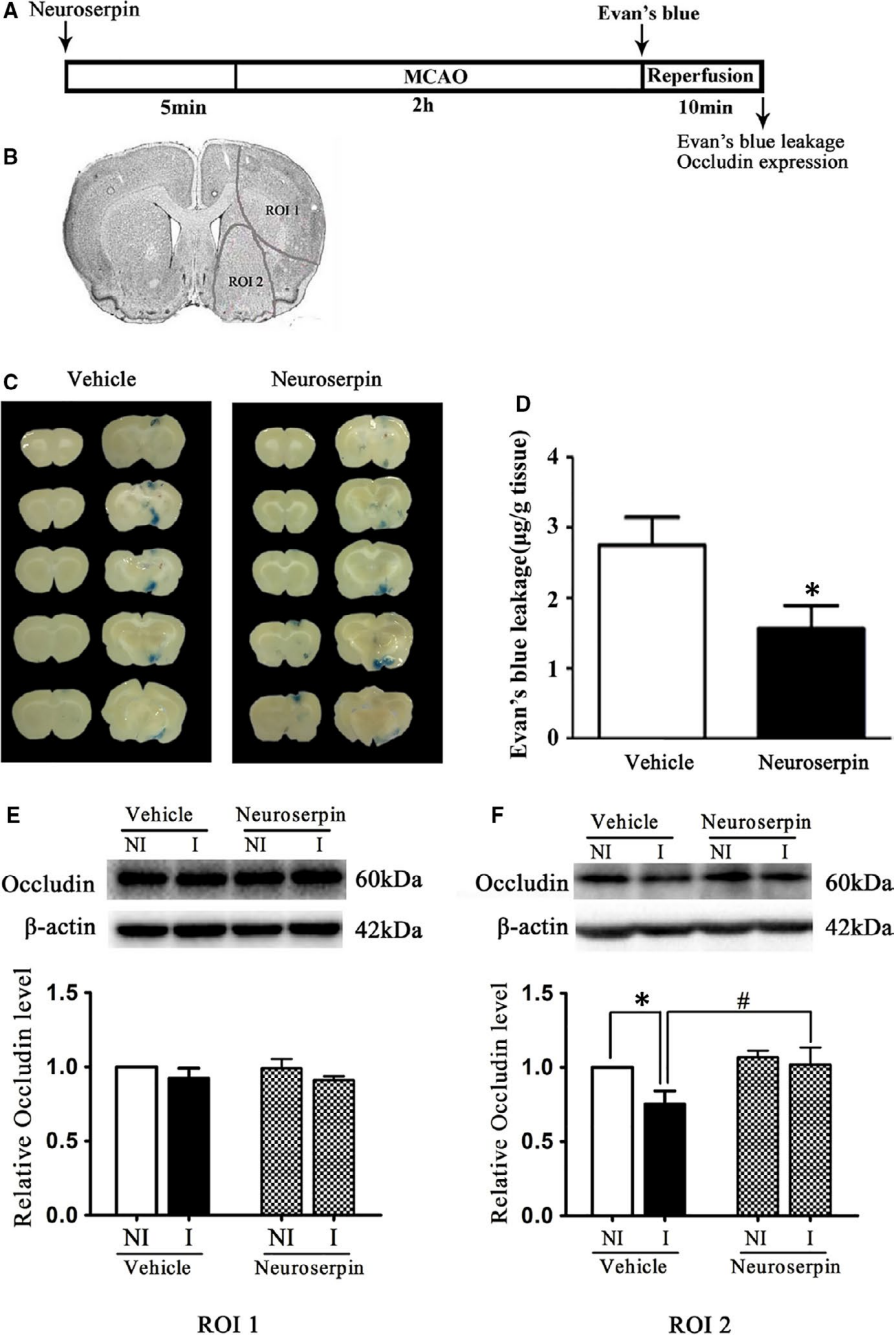
Neuroserpin treatment significantly reduced Evan's blue (EB) leakage into the ischaemic brain as well as occludin degradation after 2‐hour MCAO. A, Diagram of the experimental procedure. B, Coronal section of the rat brain, the cortical part of the grey line is the ROI 1 area region of interest (ROI) 1 (cortex and dorsal striatum), the ventral striatum and the preoptic area of the grey line are the ROI 2 area. C, Ten consecutive sections showed EB leakage from vehicle or neuroserpin‐treated rats, n = 7/group. D, EB leakage in the brain tissue was quantitated according to the external EB standard curve. EB leakage was expressed as per gram of brain tissue (μg/g). E, F, Representative Western blot images of occludin in region of interest (ROI) 1 (E) and 2 (F) in ischaemic (I) and non‐ischaemic (NI) hemisphere. Ratios of occludin (I/NI) (E) (F) were quantitated. After 2‐h MCAO, occludin was significantly decreased in ROI 2 but not ROI 1 and pretreatment with tPA inhibitor neuroserpin could prevent this increase. ^#^ *P* < 0.05 vs. Vehicle group, ^*^ *P* < 0.05 vs. ROI 1 group. (^#^ *P* < 0.05 vs. vehicle group). Data were expressed as mean ± SEM, n = 6/group

Our published paper demonstrated that 2‐hour MCAO injured brain tissue in parietal and insular cortex and dorsal striatum (ROI 1) and disrupted BBB in ventral striatum and preoptic area (ROI 2).^11^, ^12^ Recently, our results showed that inhibition of HIF‐1α alleviated BBB damage and tight junction protein occludin degradation in ROI2.^14^ In this study, we checked if ischaemia‐induced BBB damage could be reduced through inhibition of tPA activity. Neuroserpin was intra‐striatum administered as described.^24^, ^25^ EB leakage was recruited to check BBB permeability. Representative images of EB leakage in coronal brain section were shown in Figure 2C and obvious EB leakage in the ipsilateral hemisphere of brain was seen in the MCAO rats. Neuroserpin treatment significantly reduced 2‐hour MCAO‐induced BBB disruption, indicated by a significant reduction of EB leakage (Figure 2D).

We recently demonstrated that occludin degradation contributed to the BBB disruption after 2‐hour ischaemia.^12^ To determine whether the rapid disruption of occludin was due to ischaemia‐induced tPA upregulation, we next checked the effect of neuroserpin on occludin expression. Occludin was degraded in the ventral striatum and preoptic area (ROI 2, Figure 2F), but not in cortex or dorsal striatum (ROI 1, Figure 2E) and neuroserpin treatment significant decreased 2‐hour ischaemia‐induced occludin degradation (Figure 2F), indicating that 2‐hour ischaemia‐induced occludin degradation was mediated by upregulated tPA.

### 3.3 Effect of SCH23390 on 2‐hour ischaemia‐induced endogenous tPA upregulation as well as the expression of dopamine 1 receptor

**FIGURE 3:**
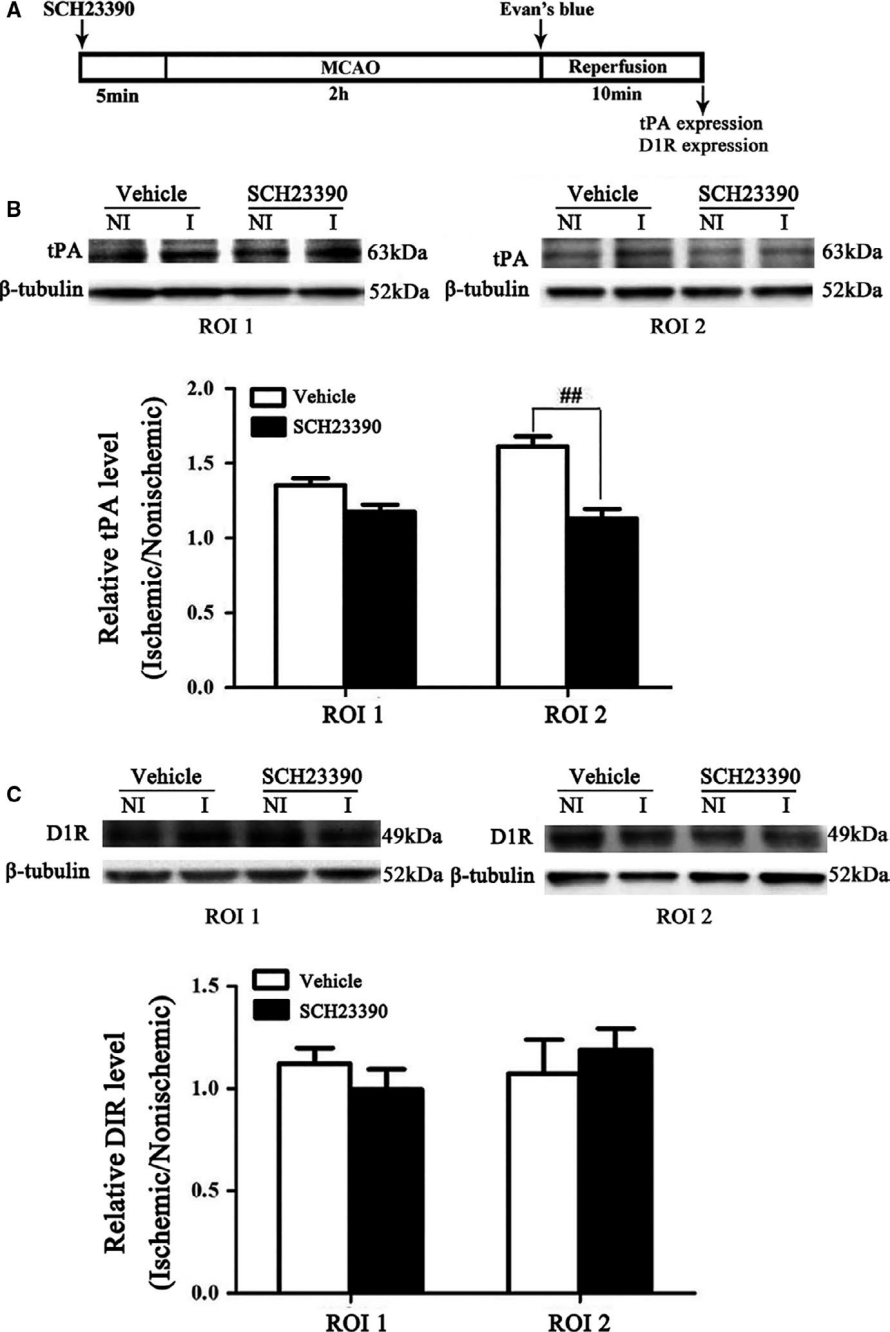
Effect of blocking D1R on 2‐h ischaemia‐induced tPA and D1R. A, Diagram of the experimental procedure. Representative Western blot revealed tPA (B) and D1 receptor (C) expression treated with SCH23390 or vehicle. The band intensity of tPA and D1R was quantitated after normalization to the β‐tublin. Two‐hour MCAO induced a significant increase of tPA level in ROI2 (right panel), but not ROI1 (left panel). Pretreatment with SCH23390 prevented tPA upregulation (*P < 0.05, compared with the ROI 1, ^#^ *P* < 0.05 vs. Vehicle group). Data were expressed as mean ± SEM, n = 5/group. C: representative Western blot revealed no significant change of D1R both ROI 1 and ROI 2 after 2‐h MCAO

D1 receptor activation has been to activate endogenous tPA in drug abuse^19^ and D1 receptor antagonist was used to block interaction of dopamine and D1 receptor. Here, we explored the effect of blocking the interaction between dopamine and D1 receptor on tPA expression. Two‐h ischaemia significantly upregulated the expression of endogenous tPA in ROI2 (Figure 3B, right panel) but not ROI 1 (Figure 3B, left panel) and D1 receptor antagonist SCH23390 significantly inhibited this effect (Figure 3B).

Ischaemic release of DA from striatum is associated with early transient changes in D1‐mediated DA neurotransmission.^17^ Here, we checked the expression of D1 receptor after 2‐hour MCAO and our results showed that no significant change was observed after 2‐hour MCAO (Figure 3C).

### 3.4 SCH23390 alleviated 2‐hour ischaemia‐induced BBB injury and loss of occludin

**FIGURE 4:**
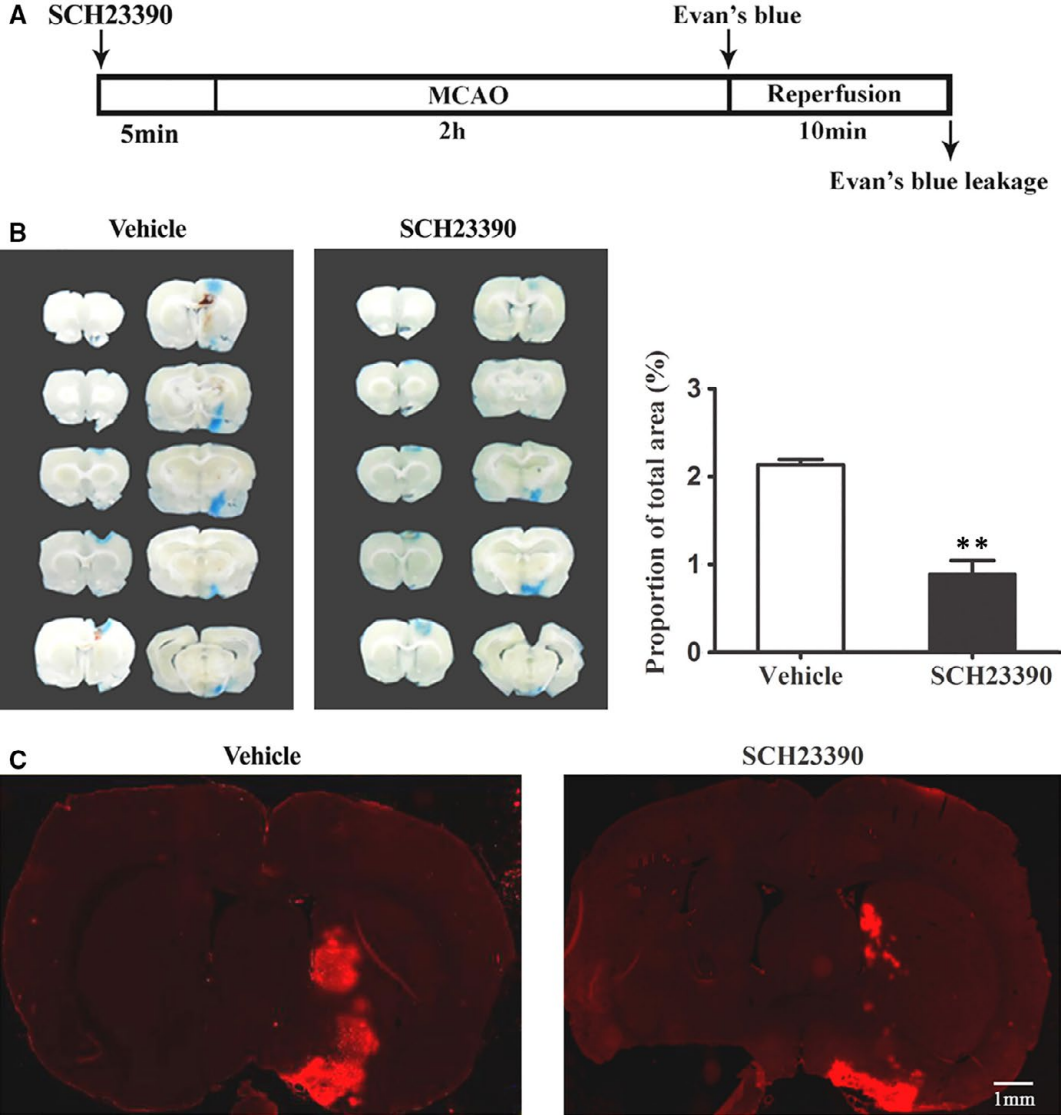
Effect of blocking D1R on 2‐h MCAO‐induced BBB disruption A, Diagram of the experimental procedure. Rats received SCH23390 5 min before the onset of 2‐h MCAO. B, Ten consecutive sections showed EB leakage from vehicle or SCH23390‐treated rats. C, EB leakage was quantitated and expressed as average area proportion of section measured (%). SCH23390 treatment significantly reduced 2‐h MCAO‐induced EB leakage (**P < 0.01, compared with the Vehicle group, n = 6/group. C, Representative representation of IgG leakage (red fluorescence). There was a significant leakage of IgG in the ventral striatum after 2‐h MCAO, and SCH23390 treatment significantly reduced ischaemia‐induced IgG leakage, n = 3/group

Dopamine has been shown to be involved in the dysfunction of the BBB,^18^ and we showed that D1 antagonist SCH23390 could inhibit 2‐hour ischaemia‐induced endogenous tPA upregulation. Therefore, we examined the effect of D1 antagonist SCH23390 on the integrity of BBB and occludin expression. Figure 4B showed the EB leakage in consecutive coronal brain slices and obvious EB leakage in the ipsilateral hemisphere of brain was seen after 2‐hour MCAO (Figure 4B) and intra‐striatum infusion of SCH23390 dramatically reduced the EB leakage proportion of the total area (Figure 4B). IgG leakage is another indicator for BBB damage.^12^ Our results demonstrated that 2‐hour ischaemia induced significant IgG leakage and SCH23390 significantly inhibited this effect (Figure 4C).

**FIGURE 5:**
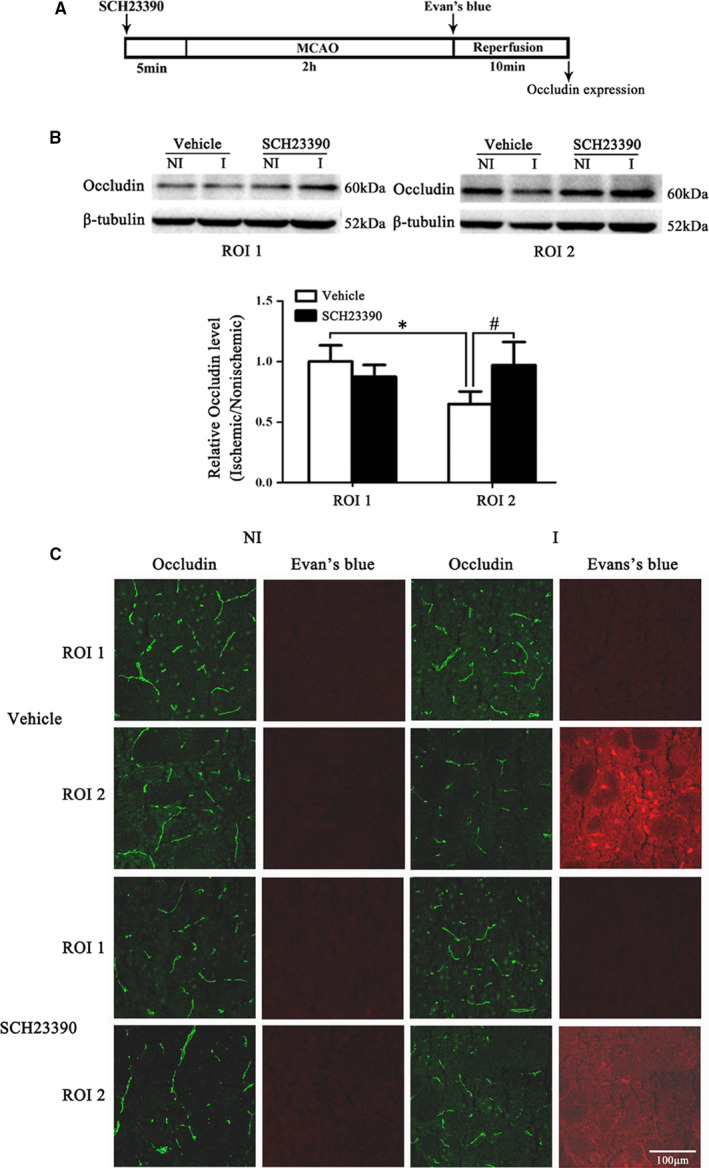
Effect of blocking D1R on 2‐h MCAO‐induced tight junction protein occludin degradation. A, Diagram of the experimental procedure. B, Western blot was used to assess occludin expression in the non‐ischaemic (NI) and ischaemic (I) hemispheric tissue. A representative Western blot revealed occludin protein expression in ROI 1 (upper panel) and ROI 2 (middle panel) treated with SCH23390 or vehicle. The band intensity of occludin was quantitated after normalization to the β‐tublin. Two‐h MCAO induced a significant decrease of occludin level in ROI 2 (*P < 0.05 vs. ROI 1), but not in ROI 1 (*P* > 0.05). Pretreatment with SCH23390 prevented occludin degradation (^#^ *P* < 0.05 vs. Vehicle group). Data were expressed as mean ± SEM, n = 5/group for Western blot. C, Representative of immunofluorescence and EB leakage of the tight junction protein occludin. Consistent with the results of Western blot, there was no significant change in the expression of occludin (green fluorescence) in the ROI 1 region, after ischaemia there is no EB leak (red fluorescence). In the ROI 2 region, the expression of occludin (green fluorescence) was significantly reduced after ischaemia, and significant EB leakage (red fluorescence) treatment with SCH23390 was effective in reducing ischaemia‐induced tight junction protein occludin degradation and EB leakage. IHC results showed occludin degradation in the area where EB leakage occurred. Pretreatment with SCH23390 significantly prevented occludin degradation as well as EB leakage. n = 3/group for IHC results. Data were expressed as mean ± SEM

To determine the effect of SCH23390 on occludin degradation, immunofluoresence and Western blot were used to detect the occludin expression. Our immunofluoresence result showed that SCH23390 treatment significantly alleviated 2‐hour MCAO‐induced occludin loss in ROI 2 (Figure 5C). Western blot data confirmed the immunofluoresence results and 2‐h ischaemia produced a significant decrease of occludin expression in ROI 2 (Figure 5B), but not ROI 1 (Figure 5B). Pretreatment with SCH23390 significantly inhibited this change (Figure 5B).

### 3.5 Effect of HIF‐1α inhibition on endogenous tPA expression

**FIGURE 6:**
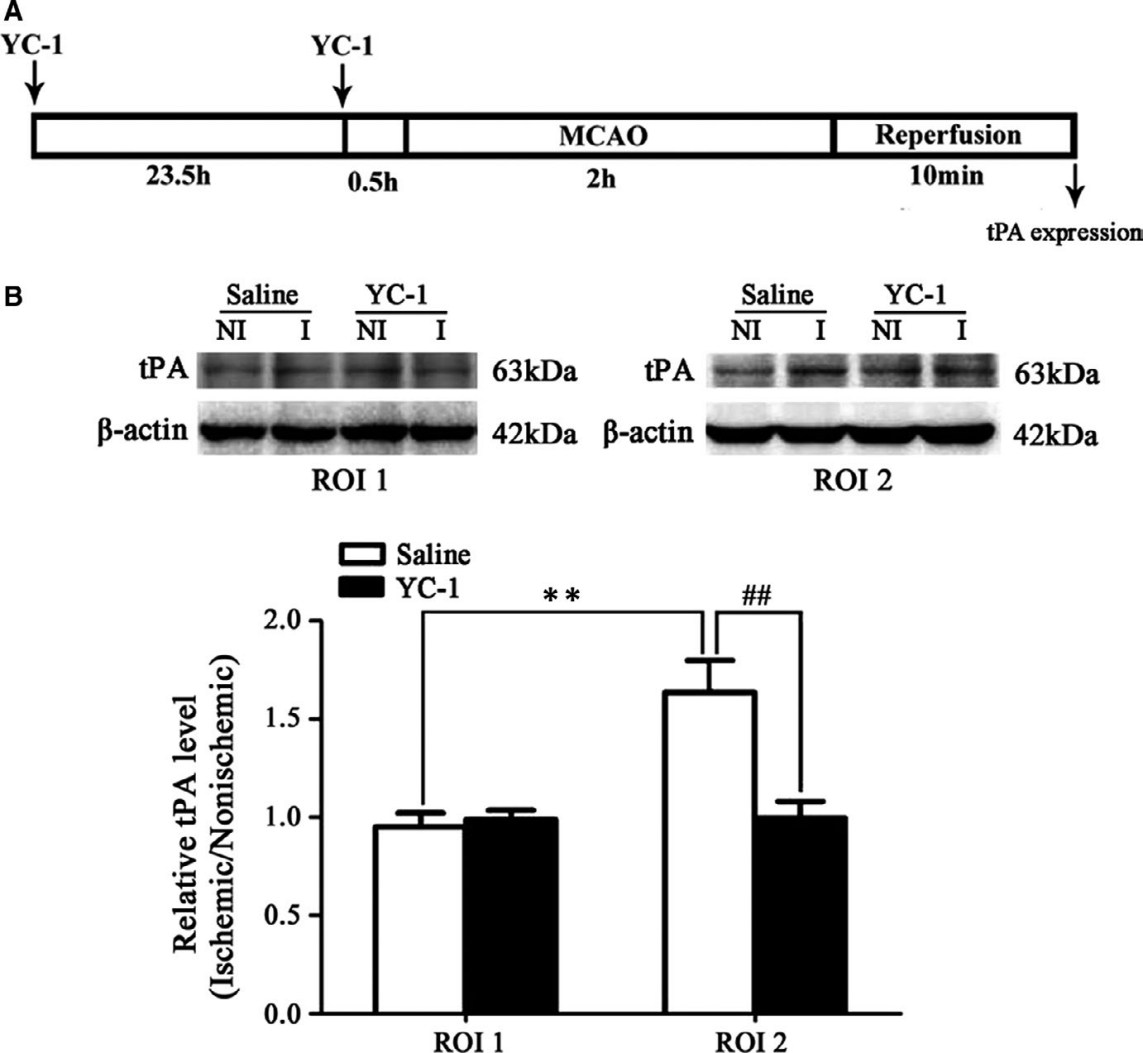
Effect of HIF‐α inhibition on tPA expression after 2‐h ischaemia. A, Diagram of the experimental procedure. Rats received YC‐1 before the onset of ischaemia. B, Western blot was conducted to detect tPA in ROI1 (left panel) and ROI2 (right panel) expression in the non‐ischaemic (NI) and ischaemic (I) hemispheric tissue. A representative Western blot revealed tPA expression treated with YC‐1 or vehicle. The band intensity of tPA was quantitated after normalization to the β‐tublin (B). Two‐h MCAO induced a significant increase of tPA level in ROI 2 (C), but not in ROI1 (B). Pretreatment with YC‐1 prevented tPA upregulation (^#^ *P* < 0.05 vs. Vehicle group). Data were expressed as mean ± SEM, n = 5/group

We recently showed that inhibition of HIF‐1α alleviated BBB damage,^13^, ^14^ reduced tight junction protein occludin degradation^14^ and inhibited MMP‐2 activity.^13^, ^14^ In addition, HIF‐1α has been shown to be a key role in the control of dopamine release.^15^ We next checked the effect of HIF‐1α inhibition on 2‐hour MCAO‐induced tPA upregulation. Our results showed that inhibition of HIF‐1α with YC‐1 significantly reduced 2‐hour MCAO‐produced tPA upregulation (Figure 6).

## 4 DISCUSSION

**FIGURE 7:**
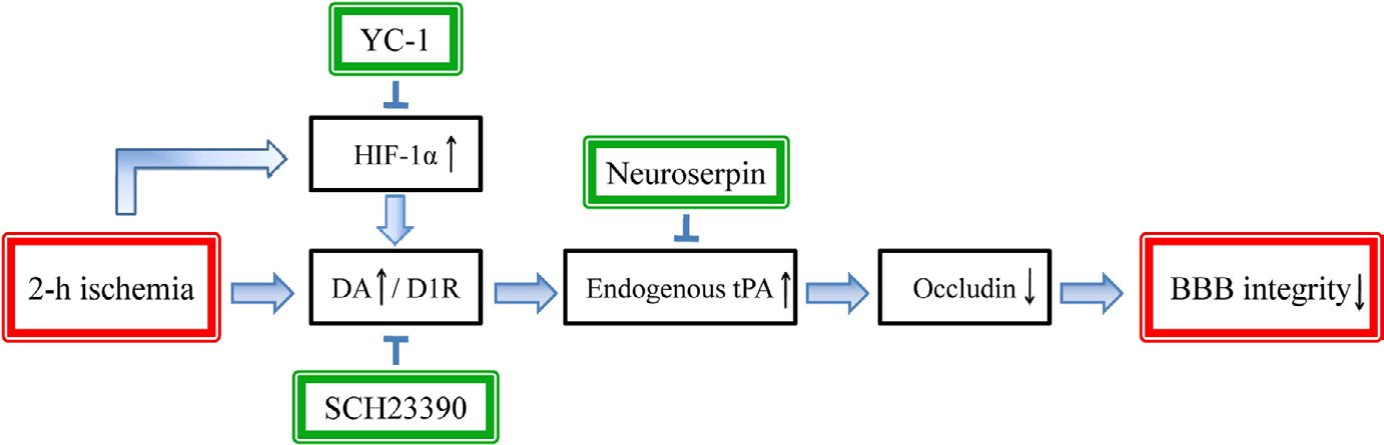
Summary. Blocking interaction of dopamine with D1 receptor reduced ischaemia‐induced HIF‐1α‐mediated BBB damage by regulating endogenous tPA during acute cerebral ischaemia

Blood‐brain barrier integrity at the time of reperfusion plays a critical role in determining the prognosis of thrombolysis with tPA and endovascular treatment.^8^, ^9^, ^30^, ^31^, ^32^ The tightness of BBB after acute ischaemic stroke is a promising target^10^, ^33^ to reduce HT in patients with intravenous tPA^8^ or post‐endovascular treatment in clinic.^9^ In this study, our result showed that (a) 2‐hour MCAO disrupted BBB in non‐infarcted striatum accompanied by endogenous tPA upregulation and inhibition of tPA with neuroserpin reduced the BBB damage; (b) D1 receptor antagonist SCH23390 significantly alleviated BBB damage by preventing tPA upregulation and occludin degradation; (c) inhibition of HIF‐1α with YC‐1 significantly decreased 2‐hour ischaemia‐induced tPA upregulation; and (d) endothelial cell contributed the major tPA secretion after 2‐hour OGD. Taken together, these observations provide strong evidence that blocking interaction of dopamine with D1 receptor reduced ischaemia‐induced HIF‐1α‐mediated BBB damage by regulating endogenous tPA during acute cerebral ischaemia (Figure 7).

Our results showed that 2‐hour ischaemia induced BBB damage accompanied by a significant increase of endogenous tPA and neuroserpin significantly reduced BBB damage, indicating that endogenous tPA plays an important role in BBB damage after acute ischaemia stroke. This is consistent with previous study showing that endogenous tPA plays an important role in the BBB damage after ischaemic stroke^2^, ^21^, ^22^ as well as pathogenesis of HT in mice^34^ and rat.^35^ Since endogenous tPA has also been demonstrated to induce BBB damage after peripheral thermal injury^36^ and traumatic brain injury^37^ and neuroserpin has been shown to reduce cerebral infarct volume, protect neurons from ischaemia‐induced apoptosis,^24^ and increase the therapeutic window for tissue‐type plasminogen activator administration in a rat model of embolic stroke,^38^ endogenous tPA could be a promising target to reduce BBB damage after various stress.

Our current results showed that endogenous tPA upregulation was found in the ventral striatum, but not in the cortex and blocking the interaction between dopamine and D1R‐reduced tPA upregulation, suggesting that ischaemia‐induced tPA upregulation was resulting from D1R activation in the striatum. It has been reported that half hour after ischaemia, dopamine reached a peak^39^ and ischaemic stroke‐induced release of DA from the striatum was greater than that from the cortex.^17^ It is worth of note that extracellular tPA activity is significantly increased by activation of the D1R pathway^19^ and tPA is involved in cholinergic interneurons excitation which is mediated by activation of D1R in the striatum.^40^ Since striatum received dopamineric projection from substantia nigra, increased endogenous tPA in striatum could be resulted from the increase of dopamine in substantia nigra and to reduce or block the effect of dopamine would be another strategy to reduce acute ischaemic stroke‐induced BBB damage.

It is well known that tight junction proteins claudin‐5, occludin and zonula occludens‐1 (ZO‐1) play critical roles in the integrity of the BBB after focal ischaemia.^41^ In our current study, we only detected the change of occludin. Because our previous study showed that after 2‐hour oxygen‐glucose deprivation (OGD), occludin was degraded by secreted MMP‐2 and claudin‐5 was redistributed from cell membrane to cytoplasm.^42^ In addition, 4‐hour OGD induced NO‐dependent claudin‐5 degradation.^33^ Furthermore, we confirmed that 2‐hour MCAO induced occludin but not claudin‐5 degradation.^12^ In our current study, duration of ischaemia is 2 hours and endogenous tPA will not disrupt the BBB through disruption claduin‐5. In addition, a recent study reported that brain‐derived endothelial cell was susceptible to OGD‐induced injury in a duration‐dependent manner as was the presence of ZO‐1 protein, cells exposed to 2 hour OGD failed to show any changes in protein, whereas 4 hour OGD led to marked decrease.^43^ In addition, another in vivo study showed that 12‐ or 24‐hour focal MCAO did not significantly change the expression of ZO‐1.^44^ So far, no study investigated the change of ZO‐1 after acute ischaemia stroke. In our current study, we aim to investigate the BBB damage within the thrombolytic time window and the duration of ischaemia is 2‐hour; therefore, the expression of tight junction protein ZO‐1 could not be affected by the related factors. An another study to investigate the expression and distribution of ZO‐1 is warranted.

Our results showed that there was no significant D1R change after 2‐hour ischaemia. Rogozinska et al reported that 1 day after stroke, D1R density decreased by 36% in the lesion core relative to sham‐operated controls, and no alterations in D1R binding were found in penumbra and other investigated regions^45^; another study demonstrated that a significant reduction in [3H] SCH23390 binding was found in the striatum from 48 hours after ischaemia.^46^ Therefore, it is unlikely that 2‐hour MCAO would induce significant change in D1R. Since after acute ischaemic stroke, dopamine release in the striatum was greater than that in the cortex,^17^ and dopamine release could be quite different between ROI1 and ROI2 after 2‐hour ischaemia.

Our results showed that D1 receptor antagonist SCH23390 significantly reduced 2‐hour MCAO‐induced BBB damage, suggesting that acute ischaemic stroke‐induced dopamine release destroyed BBB damage. Dopamine has been shown to be involved in acute 3‐nitropropionic acid‐induced striatal astrocytic cell death and dysfunction of the BBB.^18^ Our results showed that SCH23390 did not affect the expression of DR1, and according to a previous study by Yamamoto et al,^47^ the neuroprotection afforded by SCH23390 was likely mediated by blocking the interaction between dopamine and DIR.

Glutamate toxicity has been shown to play critical roles in ischaemic stroke and BBB damage. For example, blockade of NMDA or AMPA receptors could attenuate the BBB disruption in focal cerebral ischaemia, supporting a role of ionotropic glutamate receptors in BBB disruption.^48^ In addition, non‐competitive AMPA receptor antagonist perampanel affords protection against ischaemic stroke through claudin‐5‐mediated regulation of BBB permeability.^49^ Since D1 receptor toxicity may involve DARPP‐32‐dependent phosphorylation of NMDA receptor NR1,^50^ there may exist interaction between dopamine and glutamate system in BBB damage after 2‐hour ischaemia.

We have previously shown that inhibition of HIF‐1α decreased 2‐hour ischaemia‐induced BBB damage, tight junction protein occludin degradation and MMP‐2 activity^13^, ^14^; in current study, we showed that inhibition of HIF‐1α upregulation with YC‐1 alleviates 2‐hour MCAO‐induced tPA upregulation, suggesting that HIF‐1α inhibition may reduce tPA upregulation through controlling dopamine. Ischaemia has been shown to induce a significant increase of HIF‐1α accompanied by an increase of tyrosine hydroxylase expression and activity^15^ and HIF‐1α has been reported to upregulate tyrosine hydroxylase and dopamine transporter by nuclear receptor ERRγ in SH‐SY5Y cells.^51^ Of note, HIF prolyl hydroxylase inhibition has been shown to augment dopamine release in the rat brain in vivo.^52^ Combined with our results, we proposed that downregulating of HIF‐1α may inhibit tPA through regulating interaction of dopamine with D1 receptor via controlling tyrosine hydroxylase and dopamine transporter.

Plasminogen activator inhibitor (PAI‐1) could inhibit t‐PA activity; therefore, the upregulated tPA may be accompanied by decreased PAI‐1. Our unpublished data in another manuscript showed that acute ischaemia‐induced decreased expression of PAI‐1 in the brain area that showed the damage of the BBB. It has also been reported that HIF‐1α may inhibit the PA activity through stimulating the expression of PAI‐1 in normal articular chondrocytes.^53^ So, HIF‐1α may not only affect tPA’s expression but also affect tPA’s activity. Therefore, inhibition of HIF‐1α could be another strategy to reduce acute ischaemic stroke‐induced BBB damage.

One may concern about the specificity of YC‐1 as YC‐1 is also an activator of soluble guanylyl cyclase^54^ and YC‐1 has shown protective effect against white matter axons injury that is induced by nitric oxide toxicity and metabolic stress.^55^ We have discussed this issue and exclude the possibility that YC‐1 could affect the BBB integrity through modulating NO in our published paper.^13^

In summary, blocking interaction of dopamine with D1 receptor reduced ischaemia‐induced BBB damage by regulating endogenous tPA during acute cerebral ischaemia. These results extend our knowledge about the BBB damage within the thrombolysis time window and may provide new strategy and target to decrease acute ischaemia‐induced BBB damage, extend thrombolysis time window and reduce occurrence of HT.

## CONFLICT OF INTEREST

The authors declare that they have no conflict of interest.

## AUTHOR CONTRIBUTION

**Yan Wang:** Conceptualization (equal); Formal analysis (equal); Writing‐original draft (equal); Writing‐review & editing (equal). **Xiaona Wang:** Data curation (equal); Formal analysis (equal); Methodology (equal); Software (equal). **Xinyu Zhang:** Data curation (equal); Formal analysis (equal); Investigation (equal); Software (equal). **Shuang Chen:** Data curation (equal); Formal analysis (equal); Investigation (equal); Software (equal). **Yanyun Sun:** Data curation (supporting); Formal analysis (supporting); Software (supporting). **Wenlan Liu:** Writing‐review & editing (supporting). **Xinchun Jin:** Funding acquisition (equal); Investigation (equal); Project administration (equal); Validation (equal); Writing‐original draft (equal); Writing‐review & editing (equal). **Guo‐qing Zheng:** Supervision (equal); Writing‐original draft (equal); Writing‐review & editing (equal). 

## Data Availability

The data are available from the corresponding author upon reasonable request.
